# An online diagnosis method for cancer lesions based on intelligent imaging analysis

**DOI:** 10.1515/biol-2022-0625

**Published:** 2023-07-06

**Authors:** Guangliang Gu, Lijuan Shen, Xisheng Zhou

**Affiliations:** Department of Radiology, Tongxiang First People’s Hospital, Jiaxing 314500, Zhejiang, China; Department of Nuclear Medicine, Shanghai General Hospital, Shanghai Jiao Tong University School of Medicine, Shanghai 200080, Shanghai, China

**Keywords:** cancer lesion diagnosis, magnetic resonance imaging, E-hospital, diseased region location, cancer recognition

## Abstract

With the popularization and application of artificial intelligence and medical image big data in the field of medical image, the universality of modes and the rapid development of deep learning have endowed multi-mode fusion technology with great development potential. Technologies of 5G and artificial intelligence have rapidly promoted the innovation of online hospitals. To assist doctors in the remote diagnosis of cancer lesions, this article proposes a cancer localization and recognition model based on magnetic resonance images. We combine a convolution neural network with Transformer to achieve local features and global context information, which can suppress the interference of noise and background regions in magnetic resonance imaging. We design a module combining convolutional neural networks and Transformer architecture, which interactively fuses the extracted features to increase the cancer localization accuracy of magnetic resonance imaging (MRI) images. We extract tumor regions and perform feature fusion to further improve the interactive ability of features and achieve cancer recognition. Our model can achieve an accuracy of 88.65%, which means our model can locate cancer regions in MRI images and effectively identify them. Furthermore, our model can be embedded into the online hospital system by 5G technology to provide technical support for the construction of network hospitals.

## 1 Introduction

To promote people’s living conditions, strengthen health awareness, and aggravate population aging, people’s medical needs are growing. The number of patients going to a hospital for treatment is on the rise, while the space of hospitals is limited, and the outpatient volume at the time of design of many hospitals has been unable to meet the needs of hospital development [1,2]. To address this situation, many hospitals have operated in online mode to provide patients with online consultation and a series of online supporting services [3], bringing convenience to patients at the same time, reducing the offline outpatient volume, and solving the dilemma of insufficient hospital area [4].

Due to the imbalance of the medical system, it is difficult to make an accurate and perfect diagnosis of some complicated diseases in remote areas. Among them, cancer is the leading cause of death today. Early detection and early treatment are the most effective ways to deal with cancer. The wide application of computer technology has provided the necessary conditions for telemedicine for other diseases. Yuchan and Yong used sensors and applications as carriers to conduct real-time monitoring and intelligent management of chronic obstructive pulmonary disease (COPD) patients, supplemented by virtual reality equipment for respiratory rehabilitation exercise and respiratory training [5]. Huijie et al. designed a randomized controlled trial to compare the clinical efficacy and health-economic benefits of the new telemedicine mode and the traditional medical mode. It is expected to lay a foundation for the efficient utilization of medical resources and further promotion of remote diagnosis and treatment of chronic diseases [6]. In addition, Jingjie et al. believe that online hospitals should support a variety of digital transmission methods, multimedia technologies, and medical software systems, which can establish connections between different medical units [7].

Given that the diagnostic approach to cancer is biased toward meticulous screening using magnetic resonance imaging (MRI) images, there have been some specific applications. For example, Qing use MRI for the diagnosis of brain diseases and verified the superiority of MRI in the display of brain stem, basal ganglia, midline structures, skull base and cisterna, tumors in the foramen of occipital, lacunar cerebral infarction, cystic space-occupying lesions in cisterna, chronic subdural hematoma, etc. [8]. Ke et al. use MRI technology and the Voxel-Based Morphometry–Diffeomorphic Anatomical Registration Through Exponentiated Lie Algebra (VBM-DARTEL) method to conduct experiments on patients with different symptoms, and they prove the effectiveness of the DARTEL method [9]. Jiachao proposed the use of correlation analysis and the Bayesian network model to study the brain structural network of Alzheimer’s disease (AD) patients and normal people based on brain MRI data [10]. Canisius used a random forest algorithm to analyze MRI image data to achieve the diagnosis of AD. The random forest can study low-dimensional features from MRI data [11]. Wang et al. proposed an injectable amphoteric lubricant for the complete prevention of cardiac adhesion [12]. Yuan et al. proposed the phased array guided wave, which has great application prospects in plate structure monitoring and can effectively determine the location and size of damage [13]. Lu et al. have new insights into natural products that target the gut microbiome and their impact on the prevention and treatment of colon cancer [14]. Xu et al. use Atractylenolide I to enhance responsiveness to immune checkpoint blockade therapy by activating tumor antigen presentation [15]. Jing et al. proposed a related complex to improve CDK4/6 inhibition in cancer immunotherapy [16]. Chu et al. propose an emerging landscape of nanosystems based on the pre-cancer treatment microenvironment [17].

The purpose of this article is to accelerate the construction progress of online hospitals. Through the location and identification of cancer in MRI images, the patient’s condition and lesions can be quickly and accurately analyzed, to assist doctors in online complete case diagnosis. Therefore, this article proposes a cancer localization and recognition model based on MRI images to support online consultation and provide technical support for online hospital systems. The main contributions of our article are as follows:

- We propose the feature coupling unit (FCU), which can fuse features to enhance the saliency of cancer regions.
- We design an architecture of a parallel transformer to fuse the image Information and lightweight the model.
- Compared with the competitive methods, our method can achieve the 88.65% in terms of dice similarity coefficient (DSC) for localization and over 97% of accuracy.

## 2 Related work

### 2.1 Medical image segmentation

Convolutional neural networks (CNNs) have powerful segmentation capabilities in the field of medical imaging. For example, U-Net (U-shaped Network) (2015) has continuously become the most popular framework in medical image segmentation tasks since it is proposed, and people have made endless improvements to it [18]. Oktay et al. add the Attention Gate mechanism to the U-Net structure so that the target organs can obtain higher weights during training and can focus more on the segmentation of the target organs [19]. Jinfeng et al. combine two U-nets to propose a W-Net that automatically learns all the features of hepatic vessels according to the differences in the 3D structure of hepatic veins and hepatic portal veins [20]. At the same time, since most medical images are 3D data, researchers’ research on U-Net has also expanded to the 3D field. U2-Net proposed by Huang et al. used separable convolution to obtain cross-channel correlation [21]. Although the performance of the 3D network is better than that of the 2D network, it does not consider the thickness of the slice, which is also an important factor affecting the segmentation results in practical situations.

To solve the adverse effect on global modeling caused by the small receptive field of convolution operation, Chen et al. proposed a new network, TransUNet, to become a novel state-of-the-art method [22]. The TransUNet establishes the self-attention mechanism from a prediction perspective. However, Transformer also has the problem of feature resolution loss. Thus, TransUNet adopts the idea of combining CNN and transformer and adopts the U-shaped design. CNN is used for feature extraction, and then, the labeled image blocks in the extracted feature map are encoded as input sequences for extracting the global context. The encoded features are then up-sampled and combined with the previous high-resolution CNN features. Luo et al. apply the cross-teaching to semi-supervised medical images when applying CNN and Transformer [23]. Cao et al. propose Swin-Unet to feed medical images into Unet-like Transformer, which improves the segmentation of multi-organ and cardiac [24]. Heidari et al. propose HiFormer to bridge a CNN and a Transformer for medical image segmentation, which contains two multi-scale features [25]. Ban et al. proposed a 2D/3D multi-mode medical image alignment based on spatial histograms [26], and so on [27,28,29]. Qin et al. proposed an improved image fusion method based on sparse decomposition [30]. Zhao et al. introduce the latest development of pulse-coupled neural network and its application in image processing [31]. Zhang et al. proposed an improved stereo matching algorithm based on joint similarity measure and adaptive weight, which promoted the development of related techniques [32] and so on [33,34,35].

### 2.2 Medical image recognition

In the detection of AD, Sarraf and Tofighi used MRI images of diseased and normal brains for training the CNN, and the accuracy of AD detection by this model is as high as 96.5% [36]. Wang *et al.* use an improved CNN to examine AD disease, designed an eight-layer network, and improved the accuracy of the experiment to 97.65%, but the training time complexity of this method was high [37]. Weiming et al. used a CNN to identify the atrophy of the hippocampal region caused by AD. This method used the MRI images of normal people and patients in the ADNI database for training and use the method of stochastic gradient descent (SCG) to modify the parameters, through which the recognition accuracy is increased to 88% [38]. Hongmeng et al. use the improved AlexNet network to detect AD and used the data from the ADNI database and brain MRI images of patients in Beijing Tiantan Hospital, Capital Medical University for training. In the experiment, it takes less than 30 min to train the whole AlexNet network. The whole training process fully realizes the self-learning of the AlexNet network and obtains a good accuracy rate [39]. In the detection of stroke, Poole et al. use the 3D-CNN method to train and test 122 brain CT images. The experiment shows that the ROC value of this method is 99.6%, and the Recall value is 56.3%, which is a great improvement [40]. Zhang et al. focus on the intra-class variation and inter-class similarity and propose a deep learning model, which can achieve state-of-the-art performance on several datasets [41]. Cheng et al. consider the attention mechanism and design a modular group attention block to model the dependent feature between different dimensions [42]. Peng et al. analyze the two problems in medical images and propose a novel SSL model. It can apply the unlabeled data to improve recognition performance because of the adaptive threshold pseudo-labeling [43] and so on [44,45].

## 3 Cancer localization model based on feature coupling and fusion

### 3.1 Revisit

**Figure 1:**
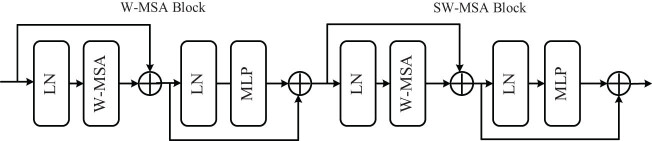
The block diagram of the Swin-transformer.

CNN [46] extracts local features by operating with surrounding pixels, which have translation invariance. However, the size of the convolution kernel and computing resources limit the performance, which results in its insufficient ability to model global information. The transformer structure benefits from the powerful global information extraction ability of its multi-head self-attention mechanism, which can build long-range dependencies between pixels. The tissues and various organs contained in MRI are clustered close together, and the boundaries of various types are similar. Effective processing of global information can bring great help to the improvement of segmentation accuracy [47,48]. Recently, the method based on the transformer structure has surpassed the method based on CNN in many visual task lists, and the Swin-transformer [49] performs particularly well. Its core module (STM) is shown in Figure 1; {z}_{i}] refers to the output features of the i] th module after MLP and residual connection. {\hat{z}}_{i}] represents the output features after concatenation of W-MSA or SW-MSA and residuals.

Each STM contains two consecutive multi-head self-attention modules. Each module consists of layer normalization (LN), multi-head self-attention layer (MHSA), residual connection, and multi-layer perceptron (MLP). The first module applies the W-MSA mechanism, and the second module adds shift-window operation (SW-MSA) based on W-MSA. The overall calculation equations (1)–(4) is as follows:

(1) {\hat{z}}_{i}={\rm{WMSA}}({\rm{LN}}({z}_{i-1}))+{z}_{i+1},]

(2) {z}_{i}={\rm{MLP}}({\rm{LN}}({\hat{z}}_{i}))+{\hat{z}}_{i},]

(3) {\hat{z}}_{i+1}={\rm{SWMSA}}({\rm{LN}}({z}_{i}))+{z}_{i},]

(4) {z}_{i+1}={\rm{MLP}}({\rm{LN}}({\hat{z}}_{i+1}))+{\hat{z}}_{i+1}.]

When calculating the MHSA mechanism, each head is calculated equation (5) as follows:

(5) {\rm{Attention}}(Q,K,V)={\rm{softmax}}\left(\frac{{{QK}}^{{\rm{T}}}}{\sqrt{D}}+B\right)V,]

where Q,{\rm{\ }}K,{\rm{V}}\in {R}^{{S}^{2}\times D}] represent the Query, Key, Value matrix, {S}^{2}] refers to the number of image patches, and D] is the number of sequential feature dimensions. The relative position encoding matrix B\in {R}^{{S}^{2}\times {S}^{2}}] , whose values are obtained in the bias matrix {R}^{(2S-1)\times (2S+1)}] .

The window-based self-attention mechanism in the Swin-transformer reduces the computational complexity of the original self-attention mechanism. The overall framework is divided into multiple stages to obtain features of different scales by referring to the hierarchical structure of the CNN base network. However, the size of medical image data sets is small, there is no common pre-training weight, and there are fewer target categories than natural images. So, the segmentation accuracy is higher, and larger models will lead to overfitting. Therefore, differing from the original Swin Transformer, the core module of our proposed cancer localization method, the FCU, interacts with features in the way of CNN and transformer fusion. The transformer structure in FCU does not need pre-training weights, and the network structure can be flexible to adjust. The transformer structure in FCU combines with CNN to supplement local location information without location coding.

### 3.2 FCU for cancer location model

**Figure 2:**
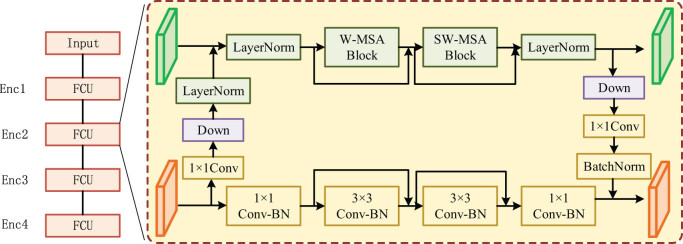
The structure of the encoder and FCU.

To enhance the awareness of network context information and retain rich detail information, this article proposes a double-branch segmentation network framework by fusing the CNN and Transformer according to the characteristics of MRI data. Its overall structure is based on the form of encoder-decoder, as shown in Figure 2.

In the encoder, it is mainly divided into four stages, namely Enc1 to Enc4 in Figure 2. First, the image of size H/2\times W/2\times C] is passed through the Stem module to extract the initial features. The stem consists of convolution, batch normalization (BN), and linear correction unit (RELU) with the size of 7 × 7 and step size of 2 whose output feature map has the size of H/2\times W/2\times C] . Then, in the Enc1 stage, the CNN branch is input into FCU after 1 × 1 convolution, and the Transformer branch is input into FCU after Patch Embed. The specific process of Patch Embed is to map the feature map to the size H/2\times W/2\times D] after 1 × 1 convolution. Then, it is expanded into the form of a sequence after the Flatten operation, and the sequence feature size is {HW}/4\times D] . Then, in Enc2 stage, the convolution branch reduces the resolution size of features from H/2\times W/2] to H/4\times W/4] by the maximum pooling layer and increases the number of channels *C* to 2*C* by 1 × 1 convolution to obtain the feature map with the size of H/4\times W/4\times 3C] . The Transformer branch converts the sequence features of size {HW}/4\times D] to {HW}/16\times 2D] by Patch Merging and then inputs them into FCU together. The subsequent processing process of Enc3 to Enc4 is like that of Enc2. The encoder contains several stages to extract shallow spatial features and deep semantic features. Its CNN branch provides local features and location information for the Transformer branch that can provide a global context for the CNN branch.

### 3.3 Information fusion module in cancer location model

**Figure 3:**
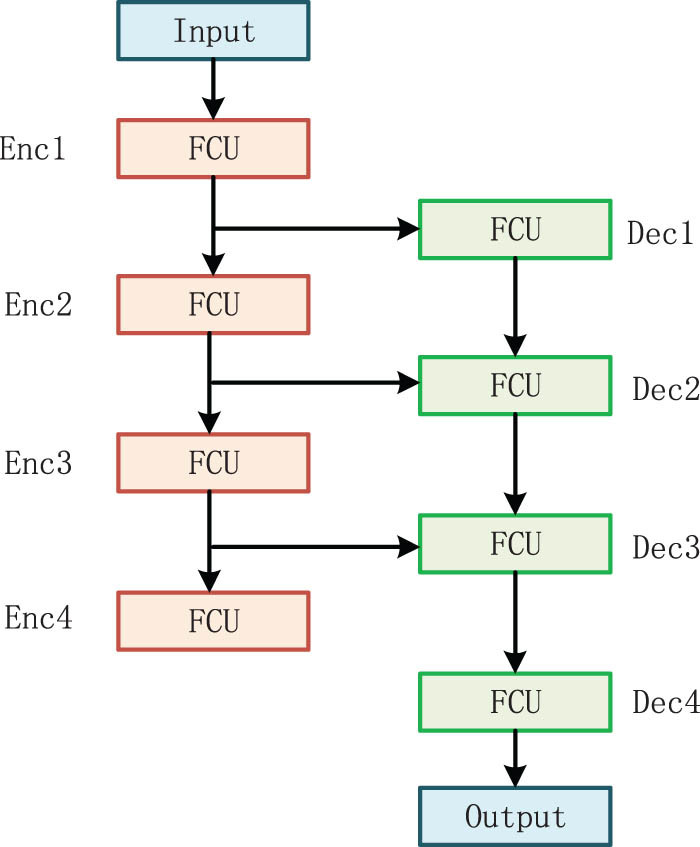
The structure of the proposed method.

**Figure 4:**
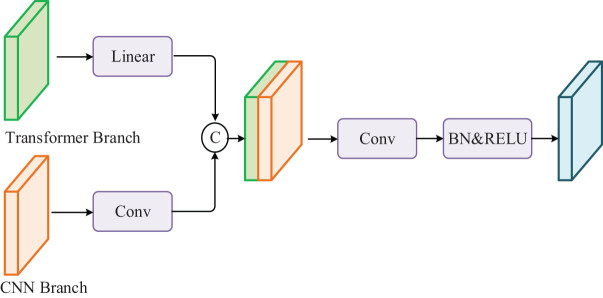
The structure of the Information fusion module.

After the encoder extracts the deep features, the dimensions and sequence features are reduced and the original input size is recovered by a decoder, which is divided into four stages, namely Dec1 to Dec4 (Figure 3). In the Dec1 stage, the CNN branch increases the resolution size of feature maps from H/16\times W/16] to H/8\times W/8] by bilinear interpolation and reduces channels from 8*C* to 4*C* by 1 × 1 convolution to obtain the feature map with a size of H/8\times W/8\times 4C] . The feature maps are concatenated by the convolutional branch in the Enc3 stage of the encoder by jump connection. And the feature map with the size of *H*/8 × *W*/8 × 8*C* is obtained after concatenation in the channel dimension (Concat). The features of *H*/8 × *W*/8 × 4*C* are obtained after reducing the number of channels to 4*C* by 1 × 1 convolution. The Transformer branch converts the sequence features of size *HW*/256 × 8*D* to *HW*/64 × 4*D* by Patch Expanding and then jumps with the sequence features output by the Transformer branch of the encoder Enc3 stage. After Concat, the sequence features of *HW*/64 × 8*D* are obtained, and then, the number of dimensions is reduced to 4*D* through the linear layer to obtain sequence features of *HW*/64 × 4*D*. The two branches then input the results of their processing into the FCU. The processing process of Dec2 and Dec3 is like that of Dec1. The output of the two branches in the stage of Dec3 goes through the final information integration module, as shown in Figure 4, and then, it is upsampled to the original image. After 1 × 1 convolution, the final segmentation prediction map is obtained, whose size is *H* × *W* × 4. To reduce the lost information when the encoder downsampling, the features after each up-sampling of the decoder and the features extracted by the encoder are fused by connection, which can improve the segmentation of MRI for multi-scale target and organ contour details.

## 4 MRI cancer recognition based on parallel transformer

### 4.1 Parallel transformer

**Figure 5:**
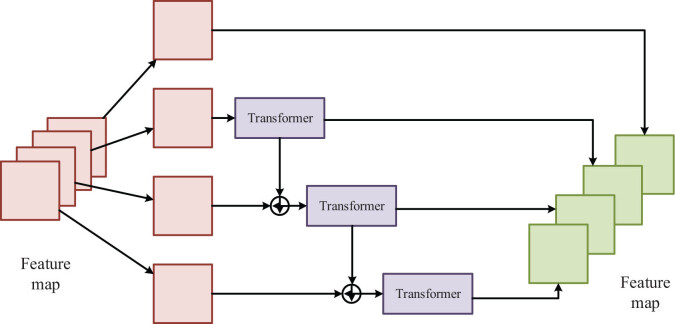
The block diagram of the hierarchical transformer.

CNN can split the input feature map according to the channel and conduct parallel computing for each group to learn features. Transformer model calculation is complex, and continuous stacking calculation can further expand the receptive field, but it lacks information at different depths. For this purpose, this article designs the parallel Transformer architecture, which is shown in Figure 5. On the one hand, the feature maps can be evenly divided into four groups via channels, and Transformer calculation is performed after grouping, which can decrease the parameters and calculation amount. On the other hand, the last set of output features is fused and input to the next layer for processing. Feature maps of different depths are spliced together, and a variety of features can be learned at the same time by increasing the receptive field, further enhancing the fusion of global information and local information.

The i] th channel is grouped into {X}_{i},\hspace{ 1em}i\in \{1,2,3,4\}] , and the output features are added to the original features of the next group after calculation to fuse the global feature information from different depths. The first set of input feature maps does not do any processing, which can save 1/4 of the number of parameters and computation and can provide original information. The second set is directly calculated, and then, we add and fuse the output to the third set of the input feature maps. So that the next neighborhood Transformer receives the global feature from the previous depth. The fourth set of input feature maps is fused with the third set of output features for calculation, and the output features of the four groups are finally spliced to obtain output features with different receptive fields.

In Transformer, different global information is joined to learn global and local features at the same time, to improve the recognition ability of cancer lesions. Transformer calculation is performed after grouping according to channels, which reduces the computational complexity. Three domain Transformers are used to form a parallel Transformer unit. By grouping the input feature maps into channels, different global features are extracted, respectively, which reduces the calculation amount and the parameters of the Transformer and maintains the light weight of the model.

### 4.2 Information fusion and lightweight

**Figure 6:**
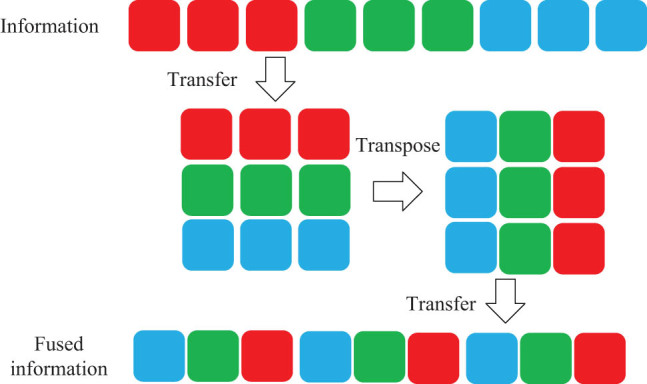
Information fusion process.

The feature map output in the parallel Transformer is directly spliced, which leads to the inability of information interaction between features of different groups. Therefore, the input features of parallel transformer units are uniformly mixed and washed according to channels by using information fusion, and the feature maps between different groups are transmitted and interacted with each other. The specific process of information fusion is shown in Figure 6. Information fusion is completed by channel grouping, transformation, transposition, and flattening of the input feature map. The advantages of this method are as follows: (1) no extra parameters and calculation amount are generated, (2) the output feature map containing global information and local information is reorganized and interacted with each other, and (3) fuse global and local information of lesions in interactive MRI images to improve model recognition ability.

To further achieve lightweight, this article designs an aggregation operation to compress the channels of output from the parallel Transformer to 1/2, and at the end, the unit aggregates all features. The aggregation operation transmits the global context information of the shallow layer to the deep layer to strengthen the shallow global lesion information transmission. The global and local lesion information of the deep and shallow layers is applied to lightweight while keeping the performance, reducing the parameters, and improving the performance of the model.

## 5 Design online hospital with our models

**Figure 7:**
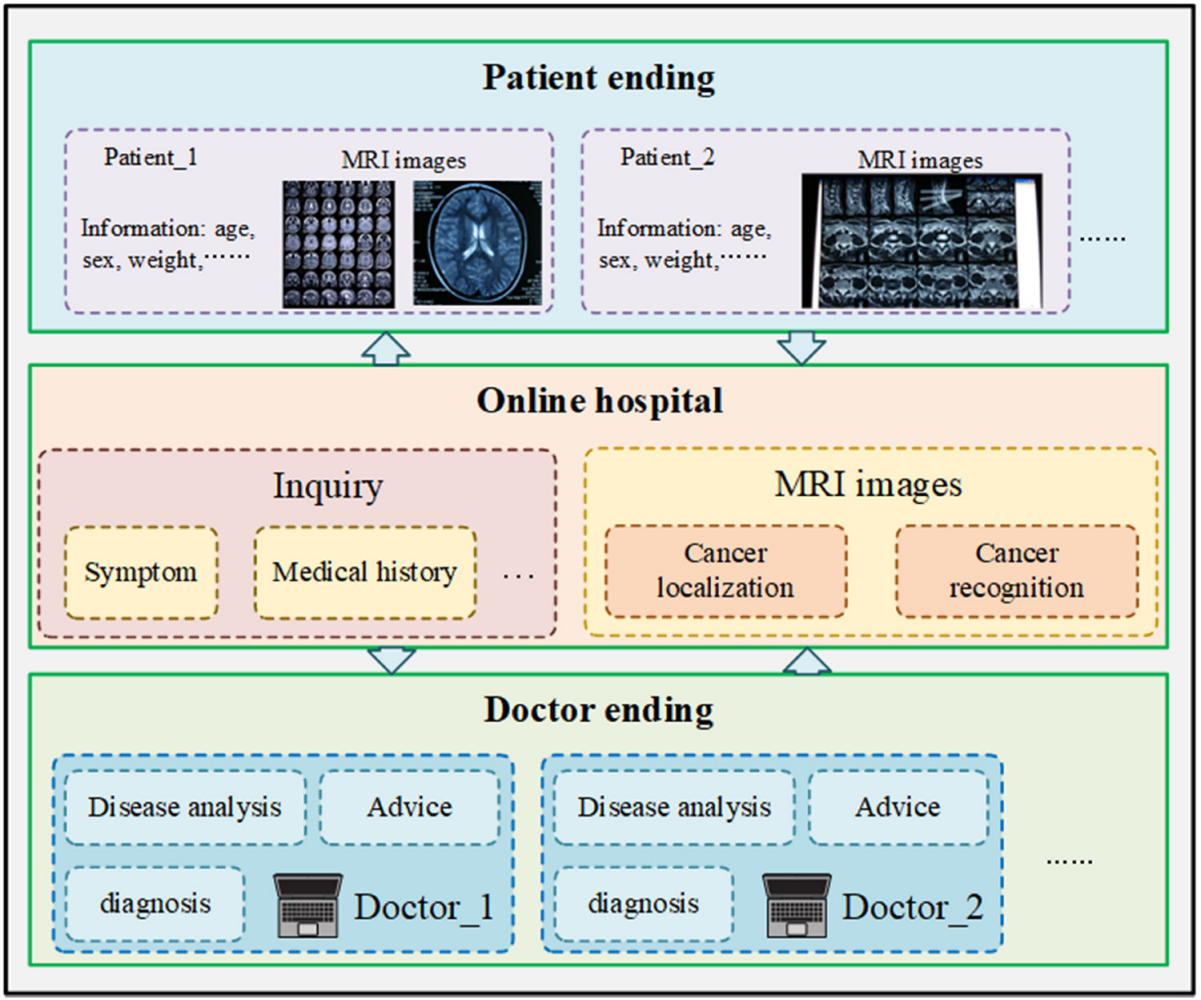
Deployment of our model for the online hospital.

The cancer localization and recognition model based on magnetic resonance images proposed in this article can accurately and quickly locate the cancer area in MRI and identify the type of cancer [32,33,34], to assist telemedicine. Therefore, we can embed our model in existing network hospitals to shorten the time for doctors to make online diagnoses and improve the medical level in remote areas. As shown in Figure 7, the system consists of the patient side, network hospital platform, and doctor side. After the online consultation service starts, the system will collect the information in real time. Then, the MRI images provided by the patients are processed and recognized by the algorithm, and the recognition results are obtained. Finally, the information is transmitted to the doctor through the network to assist the doctor to get the type and location of cancer and make the correct diagnosis.

The advantages of our system can be concluded into three points: it can be away from offline contact, avoid cross infection affected by the epidemic, increase the difficulty of patients to go to the hospital, especially for chronic diseases, and decrease the cost and difficulty of patients to seek medical treatment. The internet provides people with services, for example, epidemic prevention and popular science, remote consultation, chronic disease return consultation, and drug distribution, which undertakes and diverts the people’s medical and health needs and reduces the risk of offline population clustering of infection. To break through the geographical restrictions and expand the areas where the resources of medical are in need, more doctors prefer to open up the second stage on the Internet. In addition, the benefits of online health care are that patients can order medication in time and can also get in touch with a doctor without having to visit a hospital. For diseases that are not complicated or have more detailed examination results, online consultation can save the time of going to and from the hospital and queuing for registration, which has greater advantages.

In addition, for doctors, this system can help them write the patient’s medical records, track the patient’s lesion changes, and fully grasp the patient’s condition. The development of online hospitals can increase the number of cases seen by doctors and broaden their knowledge of the disease. It can also reduce unnecessary cases in face-to-face consultations and save valuable time. Because multiple medical experts can be consulted at the same time for the same case, it can also increase the opportunities for doctors to learn and communicate with each other. Finally, it can also slightly increase the income of doctors to some extent.

Soon, with the continuous advancement of the Internet and 5G, online medical consultation, online prescription, and drug delivery to home services will become the mainstream medical service.

## 6 Experimental and analysis

### 6.1 Dataset and analysis details

We use a prostate cancer MRI image data set (http://i2cvb.github.io/) to evaluate our model. In the data set, the MRI space size, and the number of slices for each patient are various. The height of the overall MRI ranges from 154 to 428 pixels, and the average height is 219.72 pixels. The width ranges from 154 to 512 pixels, the average width is 243.15 pixels. The number of slices ranges from 6 to 21, and the average number of slices is 10.

Experiments are carried out on the device with i7-12700 Cpu and Rtx 3090 Gpu. The operating system is Unbuntu, and we conduct our model with the Pytorch framework. The total number of rounds of the experiment is set to 1,000. Besides, we set the batch size to 16 and the initial learning rate to 0.1. After warm_up is used to warm up for one round, we adopt the decay strategy for the learning rate and the decay rate is 0.8. SGD is used as the optimizer of the model with a momentum of 0.9 and the weight decay term set to 1\times {10}^{-4}] .

To evaluate the model performance, we employ the DSC as the metric. The Dice coefficient measures how similar the segmentation labels and the predicted results are, with values ranging from 0 to 1 where 0 indicates minimum similarity and 1 indicates maximum similarity. DSC calculation equation (6) is as follows:

(6) {\rm{DSC}}=\frac{2{\rm{TP}}}{2{\rm{TP}}+{\rm{FP}}+{\rm{FN}}}]

where TP refers to the predicted pixels that are correctly classified as the target category, FP represents the predicted pixels that are misclassified, and FN represents the predicted pixels that are misclassified as non-target categories.

### 6.2 Results and discussion

**Figure 8:**
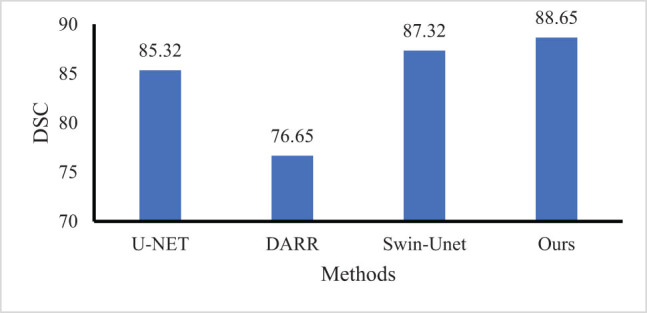
Performance comparison with other methods.

**Figure 9:**
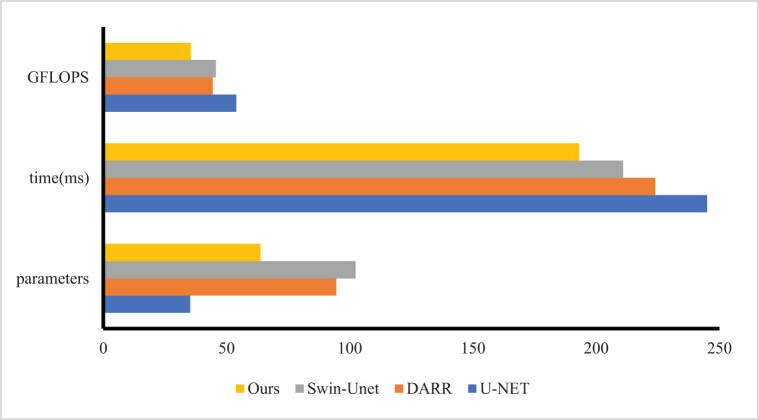
The results of parameters, cost time and GFLOPs of the methods.

Experiments are conducted on the prostate cancer MRI image data set and compared with six previous advanced technologies: U-Net, Swin-UNet and DARR. The results are shown in Figure 8. Our model achieves the best segmentation performance with an accuracy of 88.65% (DSC). Compared with SwinUNet, DSC is improved by about 1.33%. As shown in Figure 9, compared with other networks introducing attention mechanisms, such as Swin-UNet and DARR. Our model has a smaller number of parameters, faster inference time, lower computational complexity, and is more lightweight. The method based on pure CNN is not sensitive enough to the boundary information and is prone to the problem of over-segmentation. Although the method based on pure Transformer is sensitive to boundary information, it is prone to the problem of insufficient segmentation due to the loss of some features. For example, the Swin-UNet prediction of cancer appears missing, while our model predicts correctly and retains good boundary information. Experimental results show that compared with Swin-UNet and other pure CNN-based frameworks, our model pays more attention to core information and can achieve better edge prediction. For the pure Transformer approach, our model ensures both sensitivity to boundary information and prevents the loss of features.

In addition, for the classification results of cancer regions, ResNet, Deit, Conformer, and Vit are selected for comparative experiments with our model.

**Figure 10:**
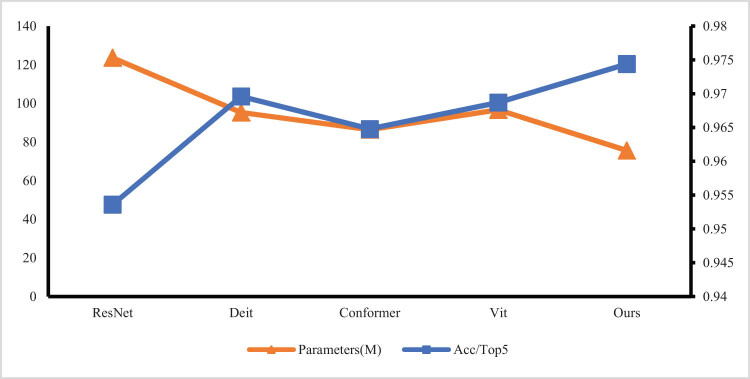
Results of parameters and accuracy of the recognition methods.

As shown in Figure 10, the experimental results show that parallel Transformer enhances the learning ability of global attention on local feature correlation, extracts multi-level feature information, improves feature interaction ability by information fusion, aggregates all global features for deep and shallow feature deep fusion, and finally improves the accuracy of the model to identify cancer.

### 6.3 Visualization results

To better illustrate the effectiveness of the cancer localization method in this article, we use some cancer samples from non-above data sets to perform cancer region segmentation with the proposed method.

**Figure 11:**
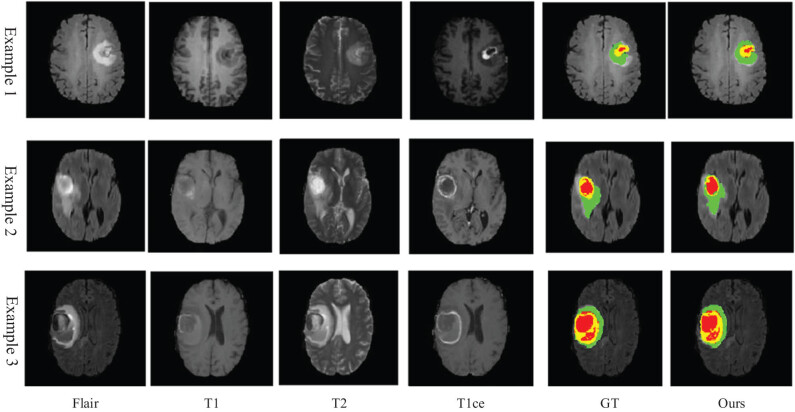
Segmentation results of brain cancer.

As shown in Figure 11, the segmentation results of our model can clearly show the three partitions of the tumor tissue, and most of the internal partition boundaries are correctly covered. The overall segmentation results of the tumor are relatively like the real label. As can be seen from Example 1, the cancer tumor shown in the MRI image is approximately circular, and its real label shows that its internal stratification is relatively clear with only a few discontinuities in the core area. The renderer generated by our model can reflect these discontinuities very accurately, but the coverage of the core area (red part) is slightly different from that of GT. In addition, the method for the outermost partition is too smooth to reflect the irregularity of the cancer edge. From Example 2, we can find the same conclusion as Example 1, our method can clearly distinguish the three partitions of the cancer tissue, but the core partition is smoother than GT, and the outermost partition fails to completely cover the tissue boundary of the cancer tumor. From Example 3, we can find that the area obtained by our method is almost the same as that obtained by GT, which is the best fit for the real situation among the three cases.

In conclusion, our cancer segmentation model can accurately segment the three partitions of tumor tissue, but it cannot achieve complete coverage in the processing of tissue edges, which may be caused by the lack of edge information caused by the deep depth of the model. However, considering that the model itself is used to assist doctors in remote diagnosis in the online hospital platform, and the lost edge information will not affect the subsequent cancer type identification; the cancer localization model proposed in this article can be used in online hospitals.

## 7 Conclusion

To provide medical resources for remote areas, we propose a cancer localization and recognition model based on magnetic resonance images with 5G technology and artificial intelligence. First, we propose the FCU to fuse the local and global features and to enhance the saliency of the cancer regions. Then, we apply the detected cancer regions to design the parallel Transformer, which can integrate the features of superficial and deep layers. Finally, we light weight our methods and apply them to the online hospital. Experiments can demonstrate that our model can accurately segment cancer regions in MRI images and identify cancer types with 88.65% of DSC and over 97% of accuracy. Meanwhile, the application of our method for the online hospital can support telemedicine. In the future, we will explore more accurate cancer detection and recognition methods, to provide more excellent data for online hospitals.
